# Identification and Characterisation of a Novel Protein FIP-sch3 from *Stachybotrys chartarum*

**DOI:** 10.1371/journal.pone.0168436

**Published:** 2016-12-20

**Authors:** Shuying Li, Leiming Zhao, Wenyi Xu, Zhonghao Jiang, Jun Kang, Fengzhong Wang, Fengjiao Xin

**Affiliations:** 1 Institute of Food Science and Technology, Chinese Academy of Agricultural Sciences, Beijing, China; 2 School of Life Sciences, Tianjin University, Tianjin, China; Columbia University, UNITED STATES

## Abstract

In this study, a novel FIP named FIP-sch3 has been identified and characterised. FIP-sch3 was identified in the ascomycete *Stachybotrys chartarum*, making it the second FIP to be identified outside the order of Basidiomycota. Recombinant FIP-sch3 (rFIP-shc3) was produced in *Escherichia coli* and purified using GST-affinity magnetic beads. The bioactive characteristics of FIP-sch3 were compared to those of well-known FIPs LZ-8 from *Ganoderma lucidum* and FIP-fve from *Flammulina velutipes*, which were produced and purified using the same method. The purified rFIP-sch3 exhibited a broad spectrum of anti-tumour activity in several types of tumour cells but had no cytotoxicity in normal human embryonic kidney 293 cells. Assays that were implemented to study these properties indicated that rFIP-sch3 significantly suppressed cell proliferation, induced apoptosis and inhibited cell migration in human lung adenocarcinoma A549 cells. The anti-tumour effects of rFIP-sch3 in A549 cells were comparable to those of rLZ-8, but they were significantly greater than those of rFIP-fve. Molecular assays that were built on real-time PCR further revealed potential mechanisms related to apoptosis and migration and that underlie phenotypic effects. These results indicate that FIP-shc3 has a unique anti-tumour bioactive profile, as do other FIPs, which provide a foundation for further studies on anti-tumour mechanisms. Importantly, this study also had convenient access to FIP-sch3 with potential human therapeutic applications.

## Introduction

Fungal immunomodulatory proteins (FIPs) are a class of small proteins that share similar sequence and structural characteristics [1]. As such, FIPs are assigned to their own family of proteins. FIP family proteins exhibit sequence identity that is greater than 57% and are made up of approximately 110 amino acid residues with a molecular weight of approximately 13 kDa. FIPs lack His, Cys and Met residues but are rich in Asp and Val residues [2]. The crystal structures of FIP-fve from *Flammulina velutipes* (PDB entry: 1OSY) [3], LZ-8 (FIP-glu) from *Ganoderma lucidum* (PDB entry: 3F3H) [4] and FIP-gmi from *G*. *microsporum* (PDB entry: 3KCW) have been resolved. FIP-fve and LZ-8 are homodimers, and FIP-gmi is a homotetramer. The FIP monomer displays an N-terminal α-helix followed by a C-terminal fibronectin III (FNIII) domain. Natural FIP protein is stabilized by hydrophobic interactions within the N-terminal α-helix. The C-terminal FNIII domain exists as an immunoglobulin-like β-sandwich fold, which is where the main biological functions take place.

FIPs engage in various biological activities. This family of proteins can agglutinate erythrocytes, regulate immunity and inhibit tumour growth [5,6]. FIPs regulate immunity by stimulating lymphocyte proliferation, inducing T_H_1-specific cytokine synthesis (interleukin-2, interferon-γ, and tumour necrosis factor β), and inhibiting anaphylaxis [1]. The anti-tumour effects that are displayed by FIPs refer to the suppression of tumour cell proliferation, promotion of tumour cell apoptosis, the induction of tumour cell cycle arrest, and the inhibition of tumour cell migration [7,8]. FIPs have very broad potential future applications in medical care, and the anti-tumour effects in particular have attracted extensive attention.

By sharing considerable sequence and structural similarity, FIPs from different sources display diverse activities. For example, FIPs from *Ganoderma spp*. have strong anti-tumour activity, while FIP-fve from *F*. *velutipes* demonstrates significant anti-allergy activity [2]. Much progress has been made in the identification of distinctive FIPs. In the early days, FIPs were primarily identified by extraction or homologous cloning methods from Macrofungi. Extracting FIPs from Macrofungi was time-consuming and tedious and resulted in a low yield. Since 1989, only seven FIPs have been identified using the extraction method: LZ-8 [6], FIP-fve [5], FIP-vvo from *Volvariella volvacea* [9], FIP-gts from *Ganoderma tsugae* [10], FIP-app from *Auricularia polytricha* [11], FIP-pcp from *Poria cocos* [12], and FIP-tvc from *Trametes versicolor* [13]. Homologous cloning involves designing primers using known FIPs as templates and cloning FIPs in the same genus, so it is limited to identifying highly homologous proteins (identity>90%), making it difficult to further characterize FIP proteins. Until now, only five FIPs have been identified using the homologous cloning method: FIP-gmi [14], FIP-gsi from *Ganoderma sinense* [15], FIP-cru from *Chroogomphis rutilus* [16], FIP-gat from *Ganoderma atrum* [7], and LZ-9 [17]. Therefore, researchers have shifted away from the extraction or homologous cloning methods for identifying new FIPs and instead search for sequence similarity in the burgeoning genome databases. Two previously unknown FIP genes were quickly identified by genome mining and were largely produced using recombinant expression [17–19]. These two FIPs were very different from previously discovered FIPs: FIP-nha was the first FIP that was identified from the ascomycete *Nectria haematococca*, and FIP-ppl from *Postia placenta* was the only basidiomycete FIP to be identified outside the order of Macrofungi.

In this study, one new FIP protein, FIP-sch3, from *Stachybotrys chartarum* was discovered using genome mining. To produce sufficient FIP-sch3 for further study, recombinant expression was used. An alignment analysis indicated that the sequence characteristics of FIP-sch3 were more similar to those of FIPs from *Ganoderma* spp. than to those of FIP-fve, so the anti-tumour properties of purified FIP-sch3 were tested. First, the anti-tumour activities of purified FIP-sch3 were analysed in five different types of human cancer cells. Then, utilizing human lung adenocarcinoma A549 as a model cell line, the purified FIP-sch3 was subjected to proliferation, apoptosis and migration assays to evaluate the anti-tumour effects. Finally, the anti-tumour mechanism of rFIP-sch3 was explored using Real-Time PCR (qPCR).

## Materials and Methods

### Cloning of recombinant FIPs (rFIPs) and the construction of the expression vector

The nucleotide sequences of LZ-8, FIP-fve and FIP-sch3 were synthesised using GenScript (Nanjing, China) and were based upon the sequences that were identified in *G*. *lucidum* (GenBank: ACD44335), *F*. *velutipes* (GenBank: ADB24832) and *S*. *chartarum* IBT 7711 (GenBank: KEY70185), respectively. To facilitate the sub-cloning of the FIP genes into the *E*. *coli* expression vector pGEX-6p-1 (Amersham Pharmacia Biotech, Buckinghamshire, UK), the following forward and reverse primers were used:

LZ8F(*Eco*RI): 5'-CCAAGAATTCTCCGACACAGC-3',

LZ8R(*Xho*I): 5'-AAAAACTCGAGGTTCCACTGAGC-3',

FveF(*Eco*RI): 5'-CCCGAATTCTCCGCTACTTC-3',

FveR(*Xho*I): 5'-AAAAACTCGAGCTTTTTCCACTC-3',

Sch3F(*Eco*RI): 5'-CCCGAATTCTCCGCTCAGAC-3',

Sch3R(*Xho*I): 5'-AAAAACTCGAGCTTCCATTGGACTAG-3'.

*Eco*RI and *Xho*I restriction sites, as underlined above, were added to the forward and reverse primers, respectively. All FIP fragments were amplified using Ex Taq DNA polymerase from Takara Biotechnology Co., Ltd (Dalian, China). The recycled PCR product was digested using restriction endonucleases (*Eco*RI and *Xho*I) and ligated into the pGEX-6p-1 vector, which was digested with the same restriction endonucleases. The ligation products were then transformed into *E*. *coli* Rosetta (DE3) competent cells (Novagen, Schwalbach/Ts., Germany). The positive transformants were selected from agar plates containing 100 μg/mL ampicillin (Amp) and were upheld by DNA sequencing. Determinations of the molecular weight (MW), isoelectric point (pI) and sequence alignment were conducted using Lasergene 7.1 software (DNASTAR, Inc., Madison, Wisconsin, USA). The phylogenetic tree was constructed using Clustalx 1.8 and MEGA 7. Statistical confidence in the phylogenetic relationships was assessed using bootstrap tests, which were replicated 1000 times.

### Expression and purification of rFIPs from *E*. *coli*

A positive Rosetta transformant (pGEX-FIP) was inoculated in Luria-Bertani (LB) medium supplemented with 100 μg/mL Amp and was incubated at 37°C overnight. Ten millilitres of the overnight cultures were diluted into 1 L of fresh LB medium containing Amp and were incubated with shaking (200 rpm) at 37°C until the optical densities (OD_600_) reached 0.6–0.8. Isopropyl-β-D- thiogalactoside (IPTG) was supplied at a final concentration of 0.1 mM to induce protein expression. After another 16 h of culture at 20°C, the cultured cells were harvested by centrifugation at 5000 × g for 10 min at 4°C; suspended in 10 mM PBS (10 mL, pH 7.2) containing 10 mM Na_2_HPO_4_, 137 mM NaCl, 2.7 mM KCl, and 2 mM KH_2_PO_4_; and disrupted by sonication. The fusion protein was purified from the cell lysate using affinity chromatography with a Glutathione Sepharose column (Pharmacia, Uppsala, Sweden). The fusion protein was treated with Prescission Protease (Shanghai PrimeGene Bio-Tech Co., LTD, Shanghai, China) at 4°C for 15–16 h, and the cleaved protein tag was removed using a Glutathione Sepharose and Superdex 75 column (10/300 GL: GE Healthcare, Fairfield, CT, USA) in PBS buffer (10 mM, pH 7.2). Endotoxins in the purified protein were removed using an Endotoxin Removal Kit (YEASEN, Shanghai, China). The purity of the resultant rFIP was evaluated using a Superdex 75 column and 15% SDS-PAGE. Protein quantification was performed using a NanoDrop-1000 Spectrophotometer (Thermo Scientific, Rockford, IL, USA).

### Cell culture

Human MCF-7 (breast cancer), A549 (lung adenocarcinoma), H520 (lung squamous cancer), HeLa (cervical carcinoma), HepG2 (liver cancer) and 293 (embryonic kidney cell) cell lines were obtained from the Cell Resource Center, Peking Union Medical College (headquarters of the National Infrastructure of Cell Line Resource, NSTI). The cell lines were confirmed by PCR and cell culture to be free of mycoplasma contamination. The species origin of each cell line was confirmed using PCR. The identity of the cell line was authenticated using STR profiling (FBI, CODIS). All the results can be viewed online (http://cellresource.cn). Cells were maintained at 37°C in a 5% CO_2_ humidified atmosphere in Dulbecco’s Modified Eagle’s Medium (DMEM) (GIBCO, Rockville, MD) containing 10% foetal bovine serum (FBS; Life Technologies, Inc., Rockville, MD), 100 units/mL penicillin, and 100 μg/mL streptomycin (Life Technologies, Inc.). Cells were trypsinized with 0.25% trypsin containing 0.04% EDTA. In all subsequent functional studies, 8 μg/mL rLZ-8 and rFIP-fve, pre-characterised FIP proteins that have been demonstrated to have direct cytotoxic effects on tumour cells [1,8], were included as positive controls.

### Cytotoxic assay

Trypsinized MCF-7, A549, H520, HeLa and HepG2 cells were adjusted to 2 × 10^4^ cells/100 μL, seeded into 96-well plates, and allowed to grow for 24 h. The media was replaced with fresh media containing 8 μg/mL rLZ-8, rFIP-fve or rFIP-sch3 and cultured at 37°C in a CO_2_ incubator for another 24 h. Images were captured using an inverted microscope (Axiovert 40 CFL, Zeiss, Germany) equipped with a digital camera (AxioCam MRm, Zeiss, Germany) to observe the morphological changes.

### Cell proliferation assay

Aliquots of 100 μL A549 and 293 cell suspension (2.5 × 10^5^ cell/mL) were seeded into 96-well plates and cultured for 24 h. One hundred microlitres of serially diluted rFIP-sch3 in DMEM without FBS were added to the plates (final concentrations of 1, 2, 4, 8, 16, 32, and 64 μg/mL), which were then continually cultured for another 24 h. After this treatment, A549 and 293 cell viability was analysed using a TransDetect^™^ Cell Counting Kit (Transgen Biotech, Beijing, China) according to the manufacturer’s instructions. The optical density (OD) was measured at 450 nm using a Multiskan MK3 (Thermo Fisher Scientific, Waltham, MA, USA). The relative cell viability was given as a percentage of OD_450_ with the PBS-treated control cells set at 100%. The inhibitory rate (%) was the result of 100 minus the virtual cell viability. The half-maximal inhibitory concentration (IC_50_) was defined as the concentration that caused 50% inhibition of cell proliferation and was calculated using SPSS 19 software (PASW statistics, IBM, New York, NY, USA).

### Tumour cell apoptosis assay

Aliquots of 1 × 10^6^ A549 cells/well were seeded into a 6-well plate and cultured for 24 h. A final concentration of 8 μg/mL of rLZ-8, rFIP-fve or rFIP-sch3 was then added. After another 24 h of culture, the apoptosis rate (percentage of apoptotic cells) was evaluated using the TransDetct^™^ Annexin V-FITC/PI Cell Apoptosis Detection Kit (Transgen Biotech, Beijing, China) with flow cytometry. Staining was conducted in accordance with the manufacturer’s protocol. The stained cells were then analysed using a flow cytometer (BD FACSCalibur, San Jose, CA, USA) with Cellquest software.

### Wound healing assay

A549 cells were cultured in 6-well plates at 5 × 10^5^ cells/well and grown in medium containing 10% FBS to a nearly confluent cell monolayer. A plastic pipette tip was used to draw a “wound” line in the cell monolayer of each well. The monolayer was subsequently washed twice with PBS to remove debris or detached cells, and 8 μg/mL rLZ-8, rFIP-fve or rFIP-sch3 was added to the plates. PBS was added to the control well as the negative control. After 24 h of incubation, the cells were washed twice with PBS. The cell morphology was observed using an inverted microscope (Axiovert 40 CFL, Zeiss, Germany), and images were taken using a digital camera (AxioCam MRm, Zeiss, Germany) coupled to the microscope (magnification, × 200). The cell motility was calculated as follows: Cell motility = (distance at 0 h—distance at 24 h) / distance at 0 h × 100%, and the distance refers to the gap area.

### Screening differentially expressed genes using qPCR

Comparisons of differentially expressed genes between samples (treated with rFIP-sch3 in 8 μg/mL) and negative controls (without rFIP-sch3 treatment) were performed using qPCR. Total RNA was isolated from approximately 2 × 10^5^ cells from each group and reverse transcribed using oligo-dT primer in a two-step quantitative RT-PCR (qPCR). Briefly, first-strand cDNA syntheses were conducted on 5 μg of total RNA using SuperScript-II RNase H-Reverse Transcriptase (Life Technologies, AB & Invitrogen, California, USA). qPCR was performed using the SYBR^®^ Green I -based method (Life Technologies, AB & Invitrogen, California, USA). The designed primer sequences are shown in S1 Table. The mRNA levels were quantified relative to endogenous Actin controls. The PCR conditions were as follows: pre-denaturation at 95°C for 2 min; 40 cycles of denaturation at 95°C for 1 min, annealing at 55°C for 1 min, and extension at 72°C for 1 min; and a final extension at 72°C for 7 min. The reactions were implemented using the real-time PCR instrument (ABI 7500, Applied Biosystems, Foster City, CA, USA). Delta CT values (CT values for genes of interest minus CT values for controls) were determined. 2-ΔΔCT was used for the relative quantification of gene expression.

### Statistical analyses

All values are presented as the mean ± SD. All experiments were performed at least in triplicate in independent experiments. Statistical comparisons were made by unpaired t tests using GraphPad Prism 5 software (GraphPad Software Inc., La Jolla, CA, USA). Differences were regarded as statistically significant for *p* < 0.05 and extremely relevant for *p* < 0.01 and *p* < 0.001.

## Results

### Sequence mining and analysis

**Fig 1:**
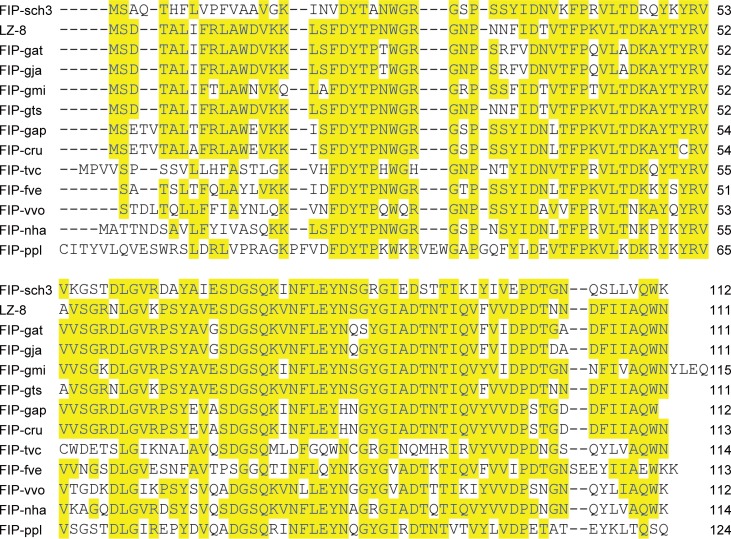
Alignment of FIP-sch3 with other FIPs. The LZ-8, FIP-gat, FIP-gja, FIP-gmi, FIP-gts, FIP-gap, FIP-cru, FIP-tvc, FIP-fve, FIP-vvo, FIP-nha, and FIP-ppl sequences came from *G*. *lucidium*, *G*. *atrum*, *G*. *japonicum*, *G*. *microsporum*, *G*. *tsugae*, *G*, *applanatum*, *C*. *rutilus*, *T*. *versicolor*, *F*. *velutipes*, *V*. *volvacea*, *N*. *haematococca*, and *P*. *placenta*, respectively. Shaded (solid bright yellow) residues match the consensus sequence exactly.

**Fig 2:**
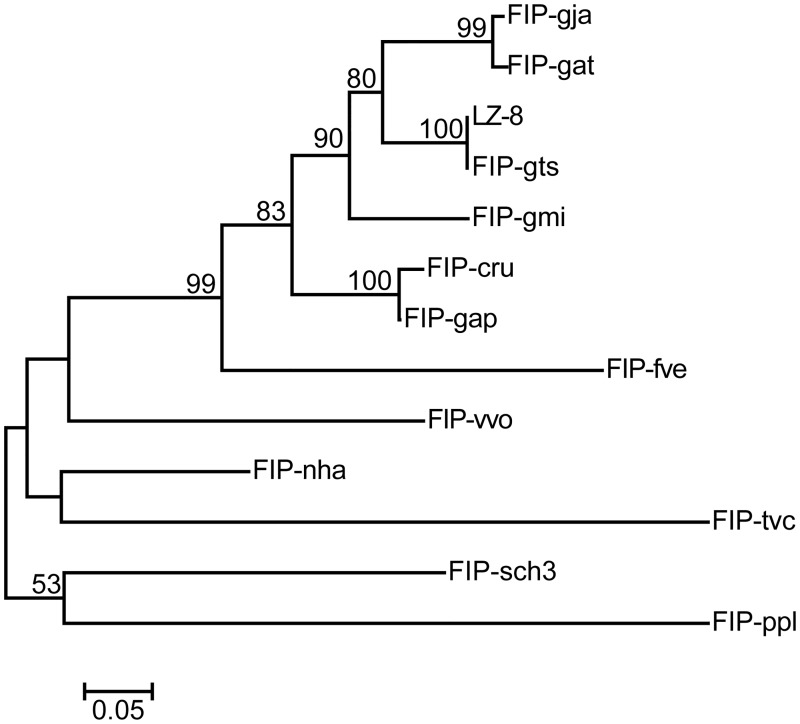
Evolutionary relationships of FIPs. The evolutionary history was inferred using the Neighbour-Joining method [21]. The optimal tree with a sum of branch length = 2.61058163 is shown. The percentage of replicate trees in which the associated FIPs clustered together in the bootstrap test (1000 replicates) are shown next to the branches [22]. The tree is drawn to scale with branch lengths in the same units as those of the evolutionary distances that were used to infer the phylogenetic tree. The evolutionary distances were computed using the JTT matrix-based method [23] and have units of the number of amino acid substitutions per site. The analysis involved 13 amino acid sequences. All positions containing gaps and missing data were eliminated. There were a total of 110 positions in the final dataset. Evolutionary analyses were conducted in MEGA7.

Searching the NCBI fungal genomic databases using the FIP-fve protein sequence revealed one small predicted protein with significant sequence similarity to FIP-fve (percent identity: 52.7%). This FIP-like sequence (GenBank ID: KEY70185) was found in the genome database for ascomycete *Stachybotrys chartarum* IBT 7711 (APIU00000000) [20]. This predicted protein, here named FIP-sch3, contains 112 amino acid residues with a calculated molecular weight of 12,568 Da and a pI of 7.93. The FIP-sch3 amino acid sequence lacks Cys, Met and His but is rich in Asp, Thr, Tyr, and Val residues with typical FIP sequence characteristics. Sequence comparisons revealed that FIP-sch3 shares significant identity with other FIPs (percent identity: 53.2% with LZ-8 and FIP-gts, 51.4% with FIP-gat and FIP-gja, 58.6% with FIP-gmi, 56.2% with FIP-cru, 55.9% with FIP-vvo, 49.5% with FIP-tvc, 61.6% with FIP-nha, 49.1% with FIP-ppl and 57.7% with FIP-gap; Fig 1). The phylogenetic tree showed that FIP-gja, FIP-gat, LZ-8, FIP-gts, FIP-gmi, FIP-cru, FIP-gap, FIP-fve, and FIP-vvo were clustered into one lineage, indicating that they are highly conserved in molecular evolution. FIP-nha and FIP-tvc, which were clustered into a separate lineage, were included in the main lineage with the above FIPs. However, FIP-sch3 and FIP-ppl clustered into separate lineages from all the above FIPs. These results indicated that FIP-sch3 had substantial phylogenetic distance from the known FIPs except for FIP-ppl, though they were likely derived from a common ancestor (Fig 2).

### rFIP expression and purification

**Fig 3:**
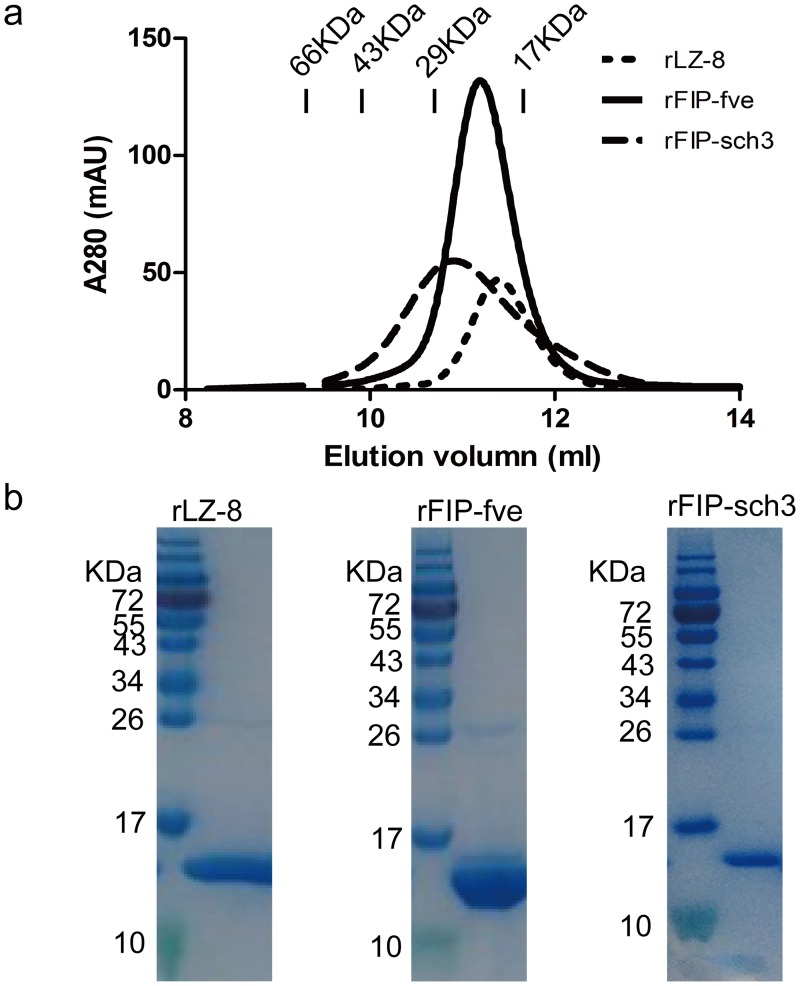
Analysis of purified rFIPs. (a) Gel filtration chromatography of rFIPs after the GST tag was removed. The standard markers noted in the figure are 66, 43, 29 and 17 kDa. Native LZ-8 and FIP-fve are dimers. The calculated molecular weight of FIP-sch3 as a dimer is 28.3 kDa. (b) SDS-PAGE results of purified rFIPs. The samples were purified using gel filtration chromatography. Purified proteins were resolved by 15% SDS-PAGE, stained with Coomassie brilliant blue R-250, and then destained with ethanol-acetic acid-water.

The FIP-sch3 gene from *S*. *chartarum* IBT 7711 (GenBank ID: KL648458) was comprised of 336 bp and ended with the stop codon TAA. The FIP-sch3 nucleotide sequence was optimized for preferred *E*. *coli* codons, synthesised and recombinantly expressed in Rosetta (pGEX-6p-1) with a GST tag at the N-terminus of the protein. The finally purified rFIP-sch3 was analysed using gel filtration chromatography with a Superdex-75 column, and the calculated average molecular weight was approximately 28.3 kDa. Because the theoretical molecular weight (containing the partial vector and restriction endonuclease sequences) of rFIP-sch3 as a monomer, a dimer, and a trimer are 14.1 kDa, 28.2 kDa, and 42.3 kDa, respectively, gel filtration results thus suggested the rFIP-sch3 was a dimer in solution. Studies have indicated that FIPs exist as dimers and are polymerized by hydrophobic interactions in the N-terminal α-helices. The purity of the non-tagged target protein was found to be greater than 98% using SDS-PAGE analysis (Fig 3). rLZ-8 and rFIP-fve were expressed, purified and analysed by the same method (Fig 3).

### Cytotoxicity of rFIP-sch3 on human cancer cells

**Fig 4:**
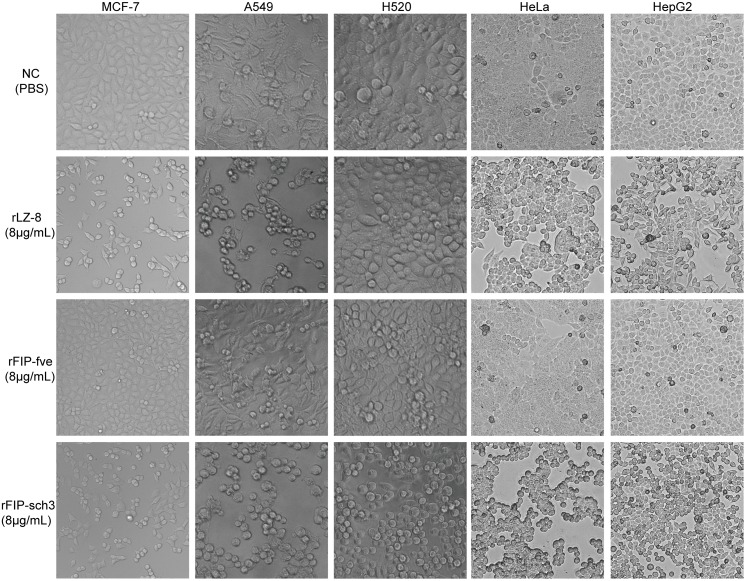
Tumour cytotoxicity assay after rFIP treatment. MCF-7, A549, H520, HeLa and HepG2 cells were treated with 8 μg/mL rLZ-8, rFIP-fve or rFIP-sch3 for 24 h; NC is the control cells after PBS treatment.

To investigate the cytotoxic effects of rFIPs on human tumour cells following 8 μg/mL rLZ-8, rFIP-fve or rFIP-sch3 treatment, the morphological changes of MCF-7, A549, H520, HeLa and HepG2 cells were monitored under an inverted microscope (Fig 4). MCF-7, A549, HeLa and HepG2, but not H520, cells that were treated with 8 μg/mL rLZ-8 for 24 h underwent apparent detachment and aggregation. After 8 μg/mL rFIP-sch3 treatment for 24 h, prominent cell shrinkage appeared in all five tumour cell lines. In contrast, the cells that were treated with FIP-fve and the untreated cells maintained their normal shapes and grew normally. These results demonstrate that rFIP-sch3 treatment is clearly toxic to all the above tumour cell lines, and rLZ-8 treatment is toxic to four of them (MCF-7, A549, HeLa and HepG2 cells), but rFIP-fve is toxic to none of the five tumour cell lines in this low dose group.

### Cytotoxicity of rFIP-sch3 in A549 cells

**Fig 5:**
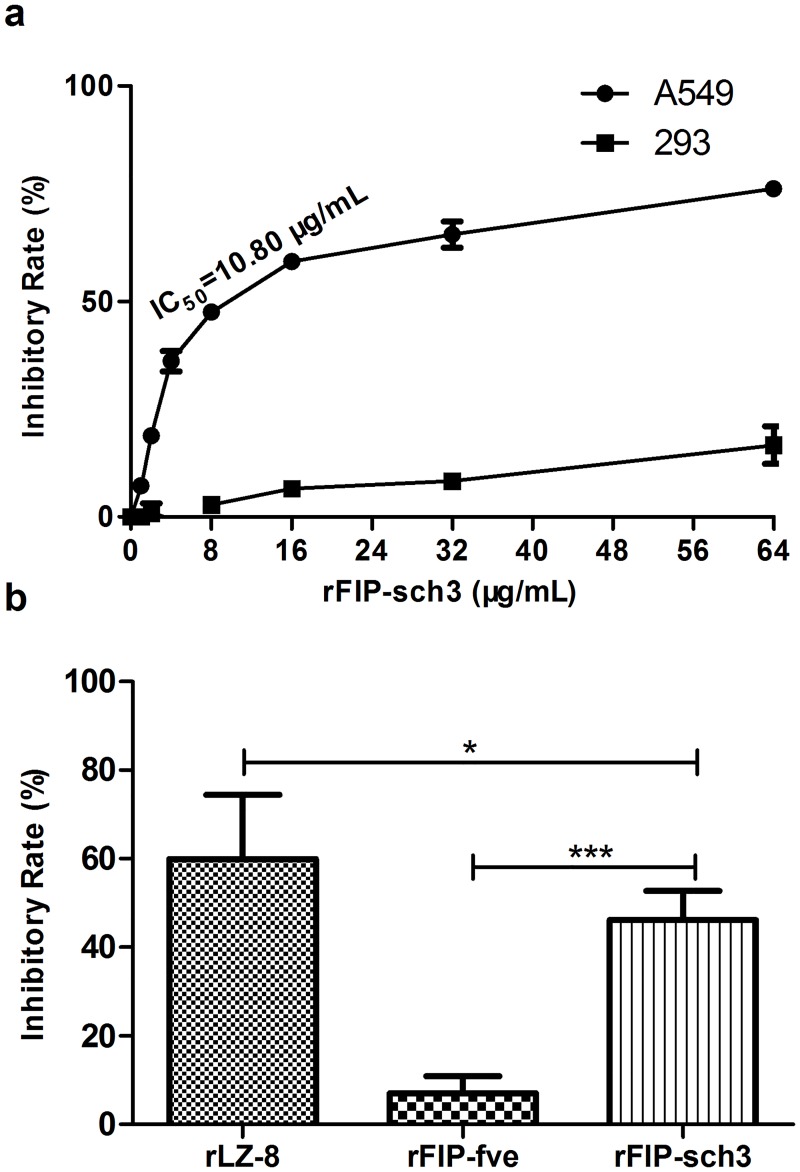
Effect of rFIP-sch3 treatment on A549 and 293 cell viability. (a) A549 and 293 cells were treated with different concentrations of rFIP-sch3 (1, 2, 4, 8, 16, 32 and 64 μg/mL) for 24 h. Cell viability was measured using a CCK assay, and the half-maximal inhibitory concentration (IC_50_) was calculated using SPSS 19 software. (b) Cytotoxicity comparison between rFIPs in A549 cells. A549 cells were treated with 8 μg/mL rFIPs for 24 h. * *p* < 0.05, and *** *p* < 0.001.

Because a remarkable amount of tumour cytotoxicity was observed in the above experiments, a CCK assay was performed *in vitro* to investigate the toxic effect of rFIP-sch3 on tumour cells. The cancer cell toxicity of rFIP-sch3 was assessed using human lung adenocarcinoma A549 cells at a final dosage of 1, 2, 4, 8, 16, 32 and 64 μg/mL. rFIP-sch3 significantly inhibited the proliferation of A549 cells at all tested concentrations (Fig 5a). At the exposure concentrations of 1 to 64 μg/mL, the inhibitory rates of rFIP-sch3 appeared to increase considerably in a dose-dependent manner. The IC_50_ values for rFIP-sch3 in A549 cells were calculated on the basis of the OD values that are shown in Fig 5a. rFIP-sch3 clearly inhibited A549 cell proliferation at an IC_50_ value of 10.80 μg/mL (Fig 5a). To exclude the drug side effects of rFIP-sch3, normal human embryonic kidney 293 cells were used to analyse the cytotoxicity of rFIP-sch3. The results indicated that rFIP-sch3 treatment nearly had no effect on the proliferation of 293 cells.

The anti-tumour activities of FIPs from different sources vary greatly. For example, the well-studied LZ-8 is strongly anti-tumourigenic, but FIP-fve is weak [1]. A comparative experiment was performed using rLZ-8 and rFIP-fve as positive controls to assess the anti-tumour activity of rFIP-sch3. To effectively observe anti-tumour effects, a final concentration of 8 μg/mL was chosen as a detection concentration in the subsequent anti-tumour assessments. The study indicated that exposure to this concentration of rLZ-8, rFIP-fve and rFIP-sch3 for 24 h resulted in inhibitory rates of 59.99%, 7.03% and 46.23%, respectively (Fig 5b). This showed that under identical experimental conditions, the cytotoxicity of rFIP-sch3 on A549 was slightly lower than that of rLZ-8 (*p*<0.05) but obviously higher than that of rFIP-fve (*p*<0.001). These results indicated that rFIP-sch3 possessed a potent anti-tumour effect on A549 cells.

### Apoptosis activity of rFIP-sch3 on A549 cells

**Fig 6:**
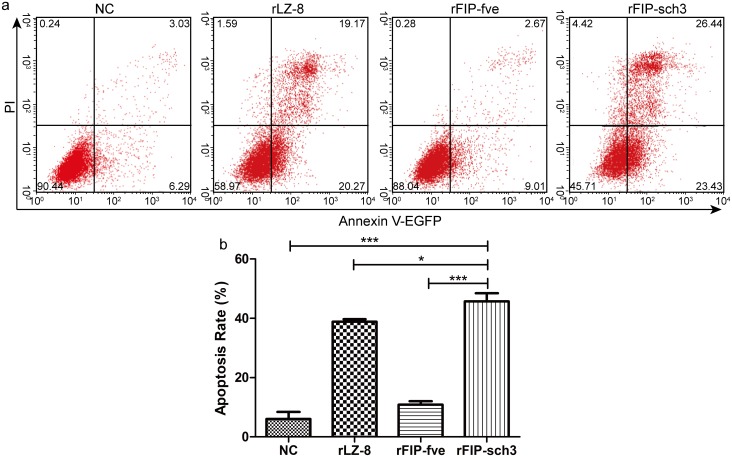
FACS analysis of apoptosis induced by rFIPs. A549 cells were subjected to treatments including PBS (negative control, NC), rLZ-8 (8 μg/mL), rFIP-fve (8 μg/mL), or rFIP-sch3 (8 μg/mL) at 37°C in a CO_2_ incubator for 24 h. The treated cells were then trypsinized, counted, and stained with annexin V-EGFP and PI, and the data were analysed using FACS. All experiments were performed in duplicate. (a) The data of one representative experiment are presented. The upper right quadrant (UR) represents late apoptotic cells that were stained with Annexin V-EGFP and PI, and the lower right quadrant (LR) represents early apoptotic cells that were stained with Annexin V-EGFP. (b) Phase percentages for the UR quadrant and LR quadrant are depicted on the bar graph. Each bar represents the mean ± SD (n = 3). *p < 0.05; ***p < 0.001, compared to the NC.

To investigate the effects of rFIPs on A549 cell viability, a flow fluorescence activated cell sorting assay was performed on A549 cells that had been treated with various rFIPs at 8 μg/mL for 24 h (Fig 6). The cells that were treated with either rLZ-8 or rFIP-sch3 had a significantly higher apoptosis rate (38.82 and 45.69%) than that of the control cells (6.10%), while the cells that were treated with rFIP-fve had a similar apoptosis rate (10.88%) to that of the control cells (Fig 6). These results demonstrated that rFIP-sch3 had an obvious effect on A549 cell viability, and the apoptotic activity in these cells was significantly greater than that in cells that had been treated with rLZ-8 (*p*<0.05) or rFIP-fve (*p*<0.001).

### Migration inhibition effect of rFIP-sch3 on A549 cells

**Fig 7:**
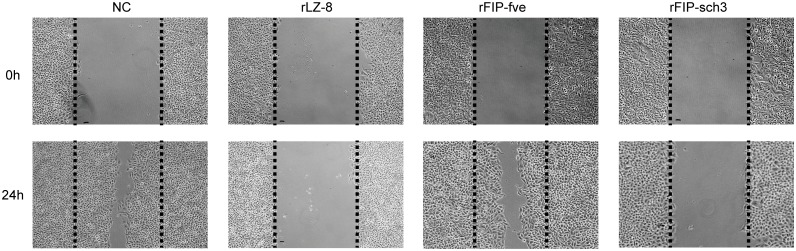
Effects of rFIPs on cell migration. A549 cells were cultured overnight and then treated with PBS (NC), rLZ-8 (8 μg/mL), rFIP-fve (8 μg/mL), or rFIP-sch3 (8 μg/mL). After incubation for 24 h, cell migration (wound healing) was monitored by microscopy.

Several studies have shown that FIPs inhibited the migration of lung cancer cells [8,14]. To understand the inhibitory effect of rFIP-sch3 on migration in A549 cells, a wound-healing assay was performed. After incubation with 8 μg/mL of rFIPs for 24 h, the cells that migrated to the denuded zone were photographed. The cells that were treated with PBS, the NC, showed acceleration of wound closure after treatment for 24 h. Cells that were treated with rLZ-8 (positive control) and rFIP-sch3 demonstrated markedly decreased wound closure activity. However, rFIP-fve, the other positive control, exhibited stronger wound closure activity that was similar to that of the NC (Fig 7). The cell motility was 84.38%, 0.67%, 82.14% and 7.14% for the NC, rLZ-8, rFIP-fve and rFIP-sch3, respectively. Compared with the controls, the cell motility of rLZ-8, rFIP-fve and rFIP-sch3-treated cells showed 99.20%, 2.05% and 91.54% reductions, respectively, at 24 h. The results demonstrated that rFIP-sch3 suppressed A549 cell migration to the denuded zone, and the migration inhibition effect of rFIP-sch3 was similar to that of rLZ-8 but markedly higher than that of rFIP-fve.

### Differentially expressed genes triggered by rFIP-sch3

**Fig 8:**
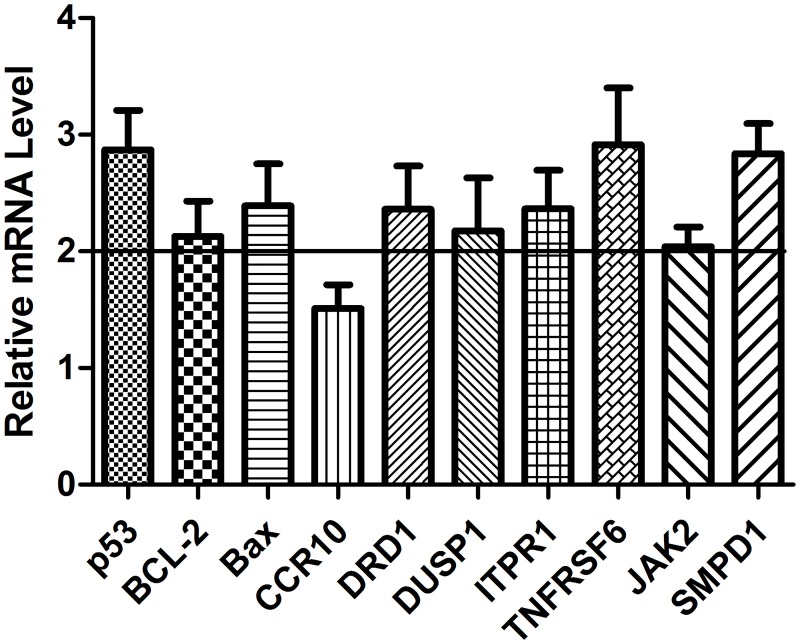
Differentially expressed gene detection resulting from rFIP-sch3 treatment. A549 cells were treated with 8 μg/mL rFIP-sch3 for 24 h. Relative mRNA levels of the ten genes (p53, BCL-2, Bax, CCR10, DRD1, DUSP1, ITPR1, TNFRSF6, JAK2 and SMPD1) were detected using qPCR, described as the means (error bars indicate SD) and represented as fold changes from controls.

In this study, 10 genes (p53, BCL-2, Bax, CCR10, DRD1, DUSP1, ITPR1, TNFRSF6, JAK2 and SMPD1) that are known to play important roles in regulating cancer cell apoptosis and migration were selected for use in detecting their differential expression using qPCR. The results showed that eight of these 10 genes were differentially expressed upon 8 μg/mL rFIP-sch3 treatment with a minimum fold change of 2, the exception being the CCR10 and JAK2 genes (Fig 8). The significantly up-regulated expression of these genes might suggest that rFIP-sch3 mediates the proliferation and metastasis of A549 cells via the apoptosis and migration pathways, which is worth further investigation.

## Discussion

In the present work, FIP-sch3, a new protein, was identified from the ascomycete *S*. *chartarum* IBT 7711 using a sequence similarity search. *S*. *chartarum* IBT 7711 is a genus of filamentous fungi that is found worldwide in soil [24]. Sequence alignments showed that FIP-sch3 displayed high sequence similarity to reported FIPs: the deduced amino acid sequence of FIP-sch3 shares 49–62% identity with other FIPs. Moreover, the phylogenetic tree showed that the evolutionary distances between FIP-sch3 and other FIPs was substantial, though all the FIPs were likely derived from a common ancestor and were strongly conserved during the process of molecular evolution. FIP-sch3 appeared to be a novel FIP. To further support this, the previously unknown FIP-sch3 was cloned, expressed, and examined for its anti-tumour activity.

FIPs have both indirect and direct inhibitory effects on cancer cells. Indirectly, FIPs activate the immune system. Of the direct inhibitory effects on cancer cells, some examples include rLZ-8 promoting apoptosis by G1/S phase arrest in human glioblastoma U-251 MG cells [25] and rFIP-gat significantly inhibiting cell growth, causing cell cycle arrest at the G1/S phase, and inducing apoptosis in MDA-MB-231 breast cancer cells [7]. Previous studies have demonstrated that rFIPs (rFIP-nha from *N*. *haematococca* and rFIP-ppl from *P*. *placenta*) that were expressed in *E*. *coli* significantly inhibited the growth of several types of cancer cells and induced apoptosis, and the anti-tumour effects were cell-specific [18, 19]. These results indicated that, despite the sequence conservation between FIPs from different sources, their anti-tumour activity varied greatly.

There have been several studies on the antineoplastic mechanisms of A549 cells (Table 1). Previous studies demonstrated that rFIP-gts inhibited A549 cell proliferation by inducing premature senescence and G1 phase arrest [26]; rFIP-gmi induced A549 cell death by activating autophagy through the Akt-mTOR-p70S6K pathway but did not induce apoptotic cell death [27,28]; rLZ-8 mediated the G1 arrest of A549 proliferation via the ribosomal protein S7-MDM2-p53 pathway [29]; FIP-fve inhibits A549 migration via RacGAP1 and suppresses the proliferation of A549 cells via the p53 activation pathway [8]. All these studies indicate that FIPs from *Ganoderma* spp. induce strong anti-tumour effects, but FIP-fve from *F*. *velutipes* exhibited a weaker effect; and their anti-tumour effects varied greatly in A549 cells.

**Table 1:** Direct anti-tumour activities of FIPs in A549 cells.

FIPs	Direct antitumour effects	References
FIP-gts from *Ganoderma tsugae*	Induces A549 premature senescence.	26
FIP-gmi from *Ganoderma microsporum*	Inhibits A549 metastasis and induces autophagy.	14 and 27
LZ-8 from *Ganoderma lucidum*	Increases A549 G1 arrest.	28
FIP-fve from *Flammulina velutipes*	Inhibits A549 migration and promotes G1 arrest.	8
FIP-sch3 from *Stachybotrys chartarum*	Induces A549 apoptosis and inhibits migration.	This study

In this study, FIP-sch3 was successfully expressed in *E*. *coli*, and the cytotoxicity assays showed that rFIP-sch3 was strongly cytotoxic in all the tumour cell lines (MCF-7, A549, H520, HeLa and HepG2 cells) but had no side effects in normal human embryonic kidney 293 cells. Further, the anti-tumour effect assays indicated that rFIP-sch3 inhibited A549 migration and proliferation by inducing apoptosis. These results also noted that the anti-tumour effects of rFIP-sch3 were similar to those of rLZ-8 but considerably greater than those of rFIP-fve. This study suggested that rFIP-sch3 is a potential anticancer agent.

Subsequently, a qPCR assay was carried out to probe the molecular mechanisms that underlie the phenotypic effects triggered by rFIP-sch3. Notably, the up-regulated expression of eight apoptosis related genes, including p53, BCL-2, Bax, DRD1, DUSP1, ITPR1, TNFRSF6 and SMPD1, suggests a mechanism for rFIP-sch3-triggered apoptosis. One mechanism of p53-mediated apoptosis is by influencing the ratio of BCL-2 (an anti-apoptotic protein) to Bax (a pro-apoptotic protein) such as to trigger the mitochondrial apoptosis pathway [30,31]. DRD1 induces breast cancer cell apoptosis by activating the DRD1/cGMP/PKG pathway [32–35]. The over-expression of DUSP1 has been reported to induce the apoptosis of NSCLC cells by inhibiting EGFR and MAPK signalling pathway [36–39]. ITPR1 plays a pivotal role in intrinsic apoptosis by mediating Ca^2+^ flux from the ER into the cytosol and mitochondria [40]. TNFRSF6, encodes a Fas receptor and mediates an apoptosis pathway in lung cancer [41,42]. SMPD1-mediated apoptosis has been suggested to predominantly involve the TNF receptor pathway [43–47]. These results suggest that rFIP-sch3 regulates various proteins that are involved in the intrinsic and extrinsic pathways, thereby inducing apoptosis.

However, the current knowledge of the above mechanisms is limited, and the molecular targets and the regulatory network for rFIP-sch3 that encompasses proliferation inhibition, apoptosis induction and migration suppression remain unclear and require further investigation. The anti-tumour mechanisms of rFIP-sch3 must be clarified to reveal potential applications. This study laid a preliminary foundation for further study of the anti-tumour mechanisms of rFIP-sch3.

## Conclusions

FIP-sch3 is a novel FIP family protein that was identified by homology search. It displayed high sequence identity (>40%) and evolutionary conservation with the previously reported FIPs. Moreover, rFIP-sch3 exhibited a broad spectrum of anti-tumour activity and obvious anti-tumour effects, such as proliferation inhibition, apoptosis induction, and migration prevention in A549 cells. In addition, the anti-tumour mechanism of rFIP-sch3 was explored. Consequently, rFIP-sch3 has the potential to serve as an anticancer agent.

## Supporting Information

S1 Table Primers for real-time PCR. (DOCX)
