# Supplementary material for: Identification and Characterisation of a Novel Protein FIP-sch3 from Stachybotrys chartarum
Source: PLoS One. 2016 Dec 20;11(12):e0168436. doi: 10.1371/journal.pone.0168436 (PMC5173029; doi:10.1371/journal.pone.0168436)
Supplement: S1 Table — (DOCX) [file pone.0168436.s001.docx]

**S1 Table: Primers for real-time PCR.**

| **Gene** | **Forward primer** | **Reverse Primer** |
| --- | --- | --- |
| Bcl-2 | GGTGGGGTCATGTGTGTGG | CGGTTCAGGTACTCAGTCATCC |
| Bax | GGGTTGTCGCCCTTTTCTA C | GGAGGAAGTCCAATGTCCAG |
| p53 | GAAGAGATGGGGGAGGGAGGCTGTCA | GCTCCGGGGACACTTTGCGTTCG |
| CCR10 | TGAAGAGGACGCATACTCGG | CCACGGTCAGGGAGACACT |
| DRD1 | GACCTTGTCTGTACTCATCTCCT | GTCACAGTTGTCTATGGTCTCAG |
| DUSP1 | ACCACCACCGTGTTCAACTTC | TGGGAGAGGTCGTAATGGGG |
| ITPR1 | GTGTCCTGCTCCACTTGAC | CCACATCTTGGCTGGTAACCAG |
| TNFRSF6 | GTACGCGGAGTGGCAGAAA | CAGAGGACGTTGCAGTAGC |
| JAK2 | TTGTGGTATTACGCCTGTGTATC | ATGCCTGGTTGACTCATCTAT |
| SMPD1 | CCGGACTCCTTTGGATGGG | TGCAGATTGGGCAGGTGAG |
| Actin | ATGGGTCAGAAGGATTCCTATGT | AAGGTCTCAAACATGATCTGGG |
